# Usefulness of Serial Multiorgan Point-of-Care Ultrasound in Acute Heart Failure: Results from a Prospective Observational Cohort

**DOI:** 10.3390/medicina58010124

**Published:** 2022-01-14

**Authors:** Marta Torres-Arrese, Gonzalo García de Casasola-Sánchez, Manuel Méndez-Bailón, Esther Montero-Hernández, Marta Cobo-Marcos, Mercedes Rivas-Lasarte, Luis Caurcel-Díaz, Pablo Rodríguez-Fuertes, Tomas Villén-Villegas, Yale Tung-Chen

**Affiliations:** 1 Department of Emergency Medicine, Hospital Universitario Fundación de Alcorcón, 28922 Madrid, Spain; martatorresarrese@gmail.com (M.T.-A.); ggcasasolaster@gmail.com (G.G.d.C.-S.); 2 Department of Internal Medicine, Hospital Clínico San Carlos, 28040 Madrid, Spain; manuelmenba@hotmail.com; 3 Department of Internal Medicine, Hospital Universitario Puerta de Hierro Majadahonda, 28222 Madrid, Spain; esthermhdez@hotmail.com; 4 Department of Cardiology, Hospital Universitario Puerta de Hierro, CIBERCV, Majadahonda, 28222 Madrid, Spain; martacobomarcos@hotmail.com (M.C.-M.); rivaslasarte@gmail.com (M.R.-L.); 5 Department of Palliative Medicine, Hospital 12 de Octubre, 28041 Madrid, Spain; luisbalbino.caurcel@salud.madrid.org; 6 Department of Emergency Medicine, Hospital Universitario La Paz, 28046 Madrid, Spain; pablo.rodriguezfuertes@gmail.com; 7 Department of Medicine, Universidad Francisco de Vitoria, 28223 Madrid, Spain; tomasvillen@gmail.com; 8 Department of Medicine, Universidad Alfonso X, 28691 Madrid, Spain

**Keywords:** acute heart failure (AHF), venous congestion, Point-of-Care Ultrasound (POCUS), VE × US (venous excess ultrasonography)

## Abstract

*Background and Objectives*: Acute heart failure (AHF) is a common disease and a cause of high morbidity and mortality, constituting a major health problem. The main purpose of this study was to determine the impact of multiorgan ultrasound in identifying pulmonary hypertension (PH), a major prognostic factor in patients admitted due to AHF, and assess whether there are significant changes in the venous excess ultrasonography (VE × US) score or femoral vein Doppler at discharge. *Materials and Methods*: Patients were evaluated with a standard protocol of lung ultrasound, echocardiography, inferior vena cava (IVC) and hepatic, portal, intra-renal and femoral vein Doppler flow patterns at admission and on the day of discharge. *Results*: Thirty patients were enrolled during November 2021. The mean age was seventy-nine years (Standard Deviation–SD 13.4). Seven patients (23.3%) had a worsening renal function during hospitalization. Regarding ultrasound findings, VE × US score was calculated at admission and at discharge, unexpectedly remaining unchanged or even worsened (21 patients, 70.0%). The area under the curve for the lung score was 83.9% (*p* = 0.008), obtaining a cutoff value of 10 that showed a sensitivity of 82.6% and a specificity of 71.4% in the identification of intermediate and high PH. It was possible to monitor significant changes between both exams on the lung score (16.5 vs. 9.3; *p* < 0.001), improvement in the hepatic vein Doppler pattern (2.4 vs. 2.1; *p* = 0.002), improvement in portal vein Doppler pattern (1.7 vs. 1.4; *p* = 0.023), without significant changes in the intra-renal vein Doppler pattern (1.70 vs. 1.57; *p* = 0.293), VE × US score (1.3 vs. 1.1; *p* = 0.501), femoral vein Doppler pattern (2.4 vs. 2.1; *p* = 0.161) and IVC collapsibility (2.0 vs. 2.1; *p* = 0.420). *Conclusions***:** Our study results suggest that performing serial multiorgan Point-of-Care ultrasound can help us to better identify high and intermediate probability of PH patients with AHF. Currently proposed multi-organ, venous Doppler scanning protocols, such as the VE × US score, should be further studied before expanding its use in AHF patients.

## 1. Introduction

Acute heart failure (AHF) is a clinical syndrome whose diagnosis is based on the probability of symptoms and signs coupled with ultrasound and laboratory markers [1]. AHF is frequently associated with high resource utilization, emergency department visits, hospital admissions. Despite this, it exhibits a non-negligible complication rate, and a post-discharge 1-year mortality that can be 25–30% with up to more than 45% deaths or readmission rates [2]. Moreover, patients with AHF with pulmonary hypertension (PH) tend to have a worse prognosis [3,4]. PH may have important consequences such as liver congestion [5], enteropathy [6], encephalopathy [7] and kidney failure [7,8].

Point-of-Care ultrasound (POCUS), performed at the bedside of the patient, allows to determine the probability of PH [1,3], based on the combination of different echocardiographic parameters; not easy to obtain in real-life practice or AHF exacerbated patients. The probability calculation is done if there are enough parameters, even if not all the parameters are available. This decreases the sensitivity and specificity of the stone. The fellowship had extensive experience and only three patients did not have all the parameters to calculate the probability of pulmonary hypertension, being only the absence of the possibility of determining the diameter of the pulmonary artery in two; so, the calculation is reliable. Given that in real life it is usually a more difficult calculation for the doctor at the bedside, other parameters that bring us closer to pulmonary hypertension are required.

Therefore, we are in need of simpler ultrasound parameters that bring us closer to determining the probability of PH and its systemic consequences [9]. Furthermore, NT-proBNP can decrease in diseases such as obesity and COPD; very prevalent in patients with pulmonary hypertension; and it has a much smaller role in the diagnosis of pulmonary hypertension than in the diagnosis of left heart failure [1,3].

Traditionally, we have relied on measuring the inferior vena cava (IVC) as a marker of central venous pressure (CVP) [10,11,12]. However, CVP does not accurately reflect the patient’s venous preload or congestion. In PH, certain valvular heart diseases, advanced chronic obstructive pulmonary disease (COPD) associated with PH or even in young and athletic patients, it is possible to find a dilated IVC without systemic congestion [13].

Currently, there is an increasing use of ultrasound parameters in the systemic congestion based on venous Doppler ultrasound, such as the hepatic and portal veins assessment [14,15,16,17,18,19,20,21,22,23,24], described more than 20 years ago, or more recent, the intra-renal vein Doppler [25,26,27,28,29,30]. Recently, it has been proven useful when integrated into a multiorgan POCUS protocol, such as the venous excess ultrasonography (VE × US) score in post-operative cardiac patients [9,31]. However, only one study regarding the portal vein [24] and two studies on the intra-renal vein Doppler [27,29] have an adequate methodology in AHF patients. To the best of our knowledge, the formulation of such an approach is in need in hospitalized AHF patients. 

## 2. Materials and Methods

This was a prospective study performed in an academic hospital, conducted in accordance with the Declaration of Helsinki, and approved by the Research Ethics Committee of our University Hospital. We obtained informed consent from each patient.

### 2.1. Patient Selection

We included patients whose main diagnosis for admission to an internal medicine and cardiology ward was AHF, at the discretion of the physician in charge. These patients had to exhibit signs attributable to congestion (any of the following: peripheral edema, ascites, jugular engorgement, crackles on pulmonary auscultation, signs of pulmonary congestion on chest X-ray), dyspnea and NT-proBNP levels > 1000 pg/mL at admission. We excluded patients < 18 years, with hemodynamic instability (vital compromise) or those who declined to participate. Moreover, if the diuretic therapy was started more than 24 h before, the patient was excluded to avoid interference with the ultrasound parameters of PH, very sensitive to decongestion.

All these patients were screened and assessed for inclusion, which was done by the study’s main investigator, who was different from the physician in charge of the patient. If after enrollment, an alternative diagnosis (such as interstitial pathology) was reached during the (ultrasound) evaluation, they were excluded from the study, and the main clinician was notified of the results. Additionally, patients who did not have a second ultrasound exam (at discharge) were excluded from the final analysis.

A sample of 30 consecutive patients who met these inclusion criteria was enrolled and prospectively studied.

### 2.2. Initial Patient Assessment

Demographic data (age, sex, weight), medical history (comorbidities, medications), risk factors for AHF (i.e., cardiopulmonary diseases), physical exam (weight, negative fluid balance), heart rate (HR), sinus rhythm/atrial fibrillation, laboratory tests (creatinine, urea, hemoglobin, white blood cells, platelets, NT-proBNP at admission and before discharge) and chest X-ray were registered.

### 2.3. Ultrasound Data Collection

Patients underwent a multiorgan ultrasound study in the first 24 h of admission and on the day of discharge.

We collected the different ultrasound parameters that could be associated with volume overload: the IVC diameter, the number of lung B-lines (Lung Score), echocardiographic findings (left ventricular diastolic diameter, left ventricular systolic diameter, interventricular septum and posterior wall thickness in diastole and systole, left ventricular ejection fraction, left and right atrial area, transmitral filling pattern, basal diameter of the right ventricle in apical plane, TAPSE, tricuspid regurgitation velocity, pulmonary artery diameter, right ventricle outflow acceleration time, pulmonary regurgitation velocity, presence of moderate or severe valvular heart disease). The VE × US protocol was measured from previously reported [9]: intra-renal Doppler ultrasound (continuous monophasic, biphasic, monophasic), portal vein Doppler ultrasound (pulsatility index (Vmax-Vmin/Vmax × 100) <30%, 30–49%, >50%) and hepatic vein Doppler ultrasound (venous flow pattern type S > D, S < D, S wave inversion). The femoral vein Doppler ultrasound was also evaluated, assessing its pulsatility (<30%, 30–50% and >50%). 

The calculation of the probability of pulmonary hypertension and the VE × US score was performed by an ultrasound fellowship-trained internal medicine physician, who had a long-standing experience in cardiac, vascular and lung US (more than 5 years). A Mindray M7 diagnostic ultrasound system with Phased Array, Curvilinear and linear transducer (Mindray España, Madrid, Spain) and a Kosmos ultrasound handheld device (EchoNous, Redmond, WA, USA) were used in the study.

The sonographer was blinded to the patient’s past medical history, vital signs, symptoms, laboratory measurements, and therapy.

### 2.4. Outcome Measures and Definitions

Our study aims to determine the impact of multiorgan ultrasound in identifying PH, a major prognostic factor in patients admitted due to AHF and assess whether there are significant changes in currently proposed multi-organ, venous Doppler scanning protocols, such as the VE × US score [9] to detect improvement in clinical (i.e., EVEREST grading score) and laboratory markers (i.e., creatinine, NT-proBNP).

The EVEREST grading score [24], as a clinical course marker of congestion during hospitalization, was calculated for each patient, at admission and at discharge.

We defined worsening renal function as a 25% increase in baseline serum creatine or an increase of 0.3 mg/dL during hospitalization [9].

PH is defined as resting mean pulmonary artery pressure of ≥25 mmHg during right heart catheterization [1]. However right heart catheterization is an invasive and technically demanding procedure that requires meticulous attention to detail to obtain clinically useful information, not always available. Therefore, in current guidelines [1] it is proposed different approaches based on ultrasound parameters and the probability of PH (low, intermediate, or high). This calculation begins in a four-chamber plane by evaluating the maximum velocity of the tricuspid regurgitation. If this is greater than 3.4 m/s, no more parameters are required to determine a high probability of pulmonary hypertension. Between 2.9 m/s and 3.4 m/ s depending on the absence or presence of other pulmonary hypertension data, an intermediate or high probability is considered. Below 2.8 m/s if there are no other pulmonary hypertension data, the probability is low and if there are, the probability is intermediate. To consider that there are other data on pulmonary hypertension, there must be alterations in two of three categories. The first category corresponds to the ventricles and assesses the ratio of the right ventricle to the left greater than 1 and the flattening of the interventricular septum. The second category corresponds to the pulmonary artery; including the measurement of the pulmonary artery (pathological above 25 mm), the velocity of pulmonary insufficiency (pathological if it is greater than 2.2 m/s) and the right ventricular outflow Doppler acceleration time (pathological if it is less than 105 ms or if there is a systolic notch. The third category is considered positive if the CVI has a diameter greater than 21 mm with collapse of less than 50% in forced inspiration or 20% in quiet inspiration or there is an increase in the size of the right atrium (pathological if it is greater than 18 cm^2^). 

Our hypothesis is that multiorgan ultrasound would help us identify AHF patients with a significant probability of PH, especially high and intermediate. Likewise, we aimed to assess whether there are significant changes in the VE × US score or femoral vein Doppler at admission and discharge.

### 2.5. Statistical Analysis

Baseline characteristics are presented as mean and standard deviation (SD) for continuous variables and count and proportions for categorical variables.

To assess normality a Shapiro–Wilk test was performed. For continuous variables that had a normal distribution, we used a Student’s t-test and the Mann–Whitney test for those who did not have normal distribution, and the ×2 or Fisher exact test for categorical variables, when it was appropriate. 

The correlations between continuous variables were tested using Spearman’s r for categorical variables. Paired sample t-tests were conducted to assess changes from admission to discharge in ultrasound parameters. The area under the curve (AUC) was calculated with the receiver operating characteristic (ROC) curve to determine the ability of the ultrasound parameters to identify patients with higher intermediate or probability of PH and kidney function worsening.

We assumed an α-value of 0.05 for two-sided hypothesis testing. Analyses were conducted with the statistical IBM SPSS software v25.0 (SPSS Inc., Chicago, IL, USA).

## 3. Results

**Figure 1:**
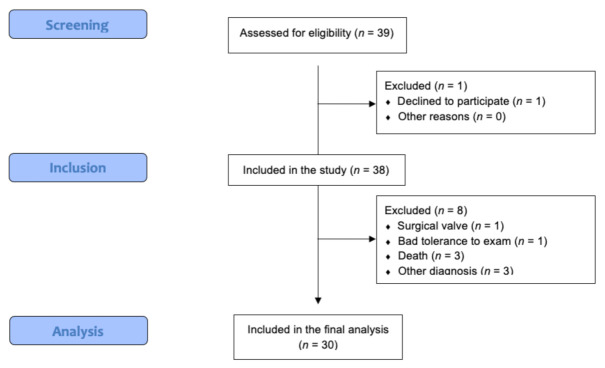
STROBE flow diagram.

During November 2021, a total of 39 patients were screened and fulfilled the inclusion criteria (summarized in Figure 1 and Table 1), one patient declined to participate and eight were excluded after inclusion: three patients died, one had a cardiac valve replacement, one could not finish the exam, and three had other diagnosis than AHF (one had diffuse lung interstitial disease and two had pneumonia, without echocardiographic signs of AHF). 30 patients were included into the final analysis.

**Figure 2:**
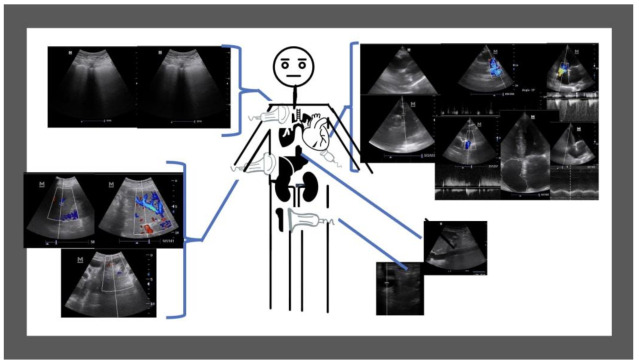
Representation of the ultrasound exam performed in all patients (*n* = 30).

The mean age was 79 years (SD 13.4) and 50% were female. Nineteen patients (63.3%) had an underlying cardiovascular illness. The mean creatinine level was 1.13 mg/dL (SD 0.5, Normal Value-NV: <0.90) and NT-proBNP was 10,846.7 pg/L (SD 11693, NV: <400) at admission. The mean creatinine level was 1.44 mg/dL (SD 0.8) and NT-proBNP was 6987.3 pg/L (SD 8999.1) at discharge. 7 patients (23.3%) had a renal function worsening. Regarding the multiorgan ultrasound exam performed (Figure 2), all patients had at least two exams performed, at admission and at discharge (see Table 1). In the paired analysis it was possible to monitor significant changes between both exams on the lung score (16.5 vs. 9.3; *p* < 0.001), improvement in the hepatic vein Doppler pattern (2.4 vs. 2.1; *p* = 0.002), improvement in portal vein Doppler pattern (1.7 vs. 1.4; *p* = 0.023), without significant changes in the intra-renal vein Doppler pattern (1.70 vs. 1.57; *p* = 0.293), VE × US score (1.3 vs. 1.1; *p* = 0.501), femoral vein Doppler pattern (2.4 vs. 2.1; *p* = 0.161) and IVC collapsibility (2.0 vs. 2.1; *p* = 0.420).

Probability of PH was calculated (9,10), resulting in a low probability in 7 patients (23.3%), intermediate in 12 (40%) and high in 11 (36.7%). VE × US score was also calculated at admission and at discharge (Table 2), with an improvement in the score in only 9 patients (30%) and unchanged or worsening in most of them (21 patients, 70.0%). The decrease in the NT-proBNP correlated with the portal vein Doppler at admission −0.440 (*p* = 0.015), portal vein Doppler at discharge −0.385 (*p* = 0.036), intra-renal vein Doppler at discharge −0.570 (*p* = 0.001) and VE × US score at discharge −0.411 (*p* = 0.024).

We calculated the EVEREST score at admission (10.1, SD 3.1) and discharge (0.74, SD 0.8). This score had a low to moderate correlation with VE × US at admission (0.532; *p* = 0.004), similar to hepatic vein (0.470; *p* = 0.011), portal vein (0.478; *p* = 0.012), intra-renal vein (0.429; *p* = 0.12) Doppler at admission. Regarding the EVEREST score at discharge, it was possible to correlate it to the VE × US score at discharge (0.461; *p* = 0.015), portal vein (0.675; *p* < 0.001), intra-renal vein (0.549; *p* = 0.003) and femoral vein Doppler (0.510; *p* = 0.007).

Regarding NYHA status, most of the patients were in a functional status of NYHA III (18 patients, 60%) at admission and NYHA I (21 patients, 70%) at discharge.

**Figure 3:**
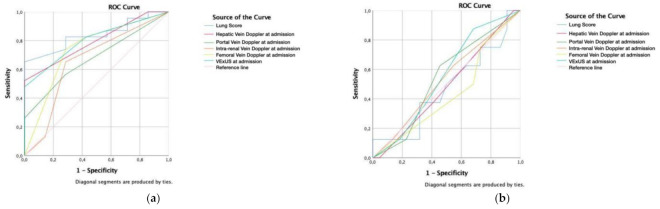
(**a**) Receiver operating characteristic (ROC) curve for predicting intermediate and high probability of Pulmonary Hypertension (PH) according to Lung Score (area under the curve-AUC-83.9%; *p* = 0.008), VE × US score (AUC 80.1%; *p* = 0.017) and hepatic vein Doppler (AUC 79.5%; *p* = 0.020) at admission; (**b**) ROC curve for predicting the probability of creatinine worsening during hospitalization, according to lung score, VE × US score, and hepatic vein Doppler at admission, without any significant results (AUC between 47.4% to 55.4%).

Receiver operating characteristic (ROC) curve was calculated for predicting intermediate and high probability of PH according to VE × US score, vein Doppler ultrasound (hepatic, portal, intra-renal and femoral), lung score (Figure 3a). The area under the curve (AUC) for the Lung Score was 83.9% (*p* = 0.008), obtaining a cutoff value of 10 that showed a sensitivity of 82.6% and a specificity of 71.4%. Followed by VE × US score (AUC 80.1%; *p* = 0.017) and Hepatic vein Doppler (AUC 79.5%; *p* = 0.020).

The ROC curve was calculated for predicting the probability of renal function worsening during hospitalization without any significant results (Figure 3b).

## 4. Discussion

The importance of venous congestion relies on its correlation with adverse events [4]. Traditionally measured with the IVC, endorsed by the European heart failure guidelines [1], however of restricted use due to its numerous limitations [13]. 

Moreover, patients with AHF with pulmonary hypertension (PH) tend to have a worse prognosis [3,4]. The evaluation of pulmonary hypertension (PH) according to these guidelines [1] should be based on assessing the ultrasound probability using 3 different categories (ventricles, pulmonary artery and IVC/right atrium), that are not always easy to obtain. 

Beaubien-Souligny proposes a venous congestion quantification system called the VE × US protocol through hepatic, portal and intra-renal venous Doppler assessment, which seems to be useful in the post-operative cardiac patient to predict kidney function worsening [9,31]. However, in AHF, there is an underestimation of creatinine at the beginning of the disease due to hemodilution, so it is not a parameter we must base therapeutic changes [1]. As seen in our cohort, there was a worsening in the creatinine results at discharge, because of hemoconcentration due to the diuretic therapy, as well as negative fluid balance.

In our study, we showed that several ultrasound parameters might be useful for monitoring the intrahospital course of the disease, such as the Lung score, hepatic and portal, vein Doppler flow. However, unexpectedly, the dynamics of the VE × US score on admission and discharge remained the same or even disimproved. This raises the question whether the VE × US protocol might be a reliable monitoring parameter to guide the adjustment of the therapy in AHF patients.

In previous studies, left heart failure is more efficiently diagnosed with the Lung score than chest X-ray and NT-proBNP [32]; comparable to our findings, Lung score and VE × US score correlated very good with an intermediate and high probability of PH, pointing out that could be a first-line diagnostic tool alternative to advanced echocardiographic studies. PH is a condition that can be underestimated by biochemical markers such as NT-proBNP, making the echocardiographic diagnosis more challenging.

Therefore, our group believes, that one of the potential roles of the VE × US protocol is aiding in the diagnosis of PH in AHF, since its diagnosis is complicated and many times we are unable to acquire all the categories to calculate its probability [3]. Our study results arise the question, whether these multiorgan Doppler assessments could serve as a new category in the diagnosis of the probability of PH.

Moreover, some exams included in the VE × US protocol, showed in isolation a good correlation with the probability of PH (i.e., the hepatic vein) or monitoring the evolution (i.e., the portal vein). This would suggest that could be reasonable to adopt different flexible approaches, rather than performing a comprehensive, time-consuming, protocol. Additionally, the hepatic veins in patients with rhythm disorders or with intracardiac devices might be difficult to interpret [33]. Therefore, selecting patients with a portal vein determination could be enough, as supported by other studies [18,19,20,24,34]. Regarding, intra-renal Doppler, some observational studies pointed out that severe congestion (monophasic pattern) was associated with an adverse prognosis in AHF [27,35]. However, obtaining adequate images can be challenging, especially in the tachypneic patient and has not been studied in patients with structural abnormalities or chronic kidney disease [36].

The femoral vein is an attractive and rapid method for the detection of PH and right ventricular failure as proposed by Denault [37] through the assessment of the pulsatility, retrograde flow and the absence of respiratory fasicity [38,39,40]; but in our study, it seems an insufficient method to be used in isolation. This could be in part that our patients could not be positioned in supine ulna (orthopnea), as described in previous studies. As in our study, we found that the femoral vein could have a similar role as the IVC.

To the best of our knowledge, our study is the first to assess the evolution of the different multiorgan venous Doppler flows in patients with AHF.

Moreover, as showed in previous studies [21] and reproduced here, an important proportion of patients admitted with AHF remained with ultrasound markers of congestion at discharge despite improvement in symptoms. This probably might help identifying patients that are particularly vulnerable to re-admission or death from AHF (as a sign of severity). Considering this information could be in fact be useful for monitoring, such as close outpatient follow-up to avoid exacerbations, therapy adjustment, etc.

We acknowledge some study limitations. First, a small sample of AHF patients, the expert sonographer performed all ultrasound scans on patients consecutively admitted to the internal medicine and cardiology department, which limits the generalizability of our results, and ought to be validated in future studies. This same sonographer was in charge of the PH probability adjudication. Second, our study did not analyze the dynamic changes according to the therapy received in different stages, and the patient outcomes, which would have a higher clinical impact. Third, because the time required to perform a whole exam makes it difficult to repeat it daily, which its use could be further studied.

Also, the hepatic vein Doppler assessment was not integrated with concurrent electrocardiogram tracing due to unavailability in our machine; which is common in most of the non-cardiology medical departments; although many of our patients had telemetry monitoring, which facilitated interpretation.

Another limitation is the poor performance of VE × US (and any other ultrasound parameters) in predicting the renal function worsening, therefore the results from this study provide insights on the need to search other meaningful outcomes, and an opportunity to further investigate the role of ultrasound in this prevalent disease.

A strength of our real-life practice study is that, as expected, the cohort is heterogeneous, which allows us to emphasize the fact that VE × US may not be applicable to all patients with AHF, as suggested by previous studies. We suggest that it could be of use in the assessment of patients with PH. More studies are needed to define the population to which might be useful to perform this protocol.

Therefore, for this purpose, we suggest the study can be considered hypothesis-generating and the conclusions must be contrasted with larger studies.

## 5. Conclusions

In conclusion, our study suggests that performing serial multiorgan point-of-care ultrasound can help us to better identify high and intermediate probability of pulmonary hypertension patients with acute heart failure. Currently proposed multi-organ, venous Doppler scanning protocols, such as the VE × US score, should be further studied before expanding its use in AHF patients.

## Figures and Tables

**Table 1:** Demographics and clinical characteristics of patients included (*n* = 30).

Demographics	*n* (%)
Gender (female)–*n* (%)	15 (50.0)
Age (years)–mean (SD)	79 (13.4)
**Previolus Diseases**	***n* (%)**
Hypertension–*n* (%)	25 (83.3)
Dyslipidemia–*n* (%)	12 (40.0)
Diabetes mellitus–n (%)	8 (26.7)
Chronic kidney disease (stage 3 or later)–*n* (%)	8 (26.7)
Previous recent hospitalization–*n* (%)	5 (6.7)
Cardiovascular disease–*n* (%)	19 (63.3)
Atrial fibrillation–*n* (%)	8 (26.7)
Reduced ejection fraction–*n* (%)	3 (10.0)
Pulmonary disease–*n* (%)	9 (30.0)
**Physical Exam**	**mean (SD)**
Weight (kg) at admission–mean (SD)	81.1 (16.7)
Weight (kg) at discharge–mean (SD)	65.6 (19.2)
**Laboratory Results**	
NT-proBNP at admission pg/L–mean (SD)	10,846.7 (11,693.8)
Urea at admission mg/dL–mean (SD)	58.3 (29.2)
Sodium at admission mg/dL–mean (SD)	137.5 (6.5)
Creatinine at admission–mg/dL–mean (SD)	1.13 (0.5)
Hemoglobin at admission–g/dL–mean (SD)	12.9 (2.4)
NT-proBNP at discharge pg/L–mean (SD)	6987.3 (8999.1)
Urea at discharge mg/dL–mean (SD)	88.7 (41.1)
Sodium at discharge mg/dL–mean (SD)	141.1 (3.9)
Creatinine at discharge–mg/dL–mean (SD)	1.3 (0.5)
Hemoglobin at discharge–g/dL–mean (SD)	12.9 (2.2)
Change in the NT-proBNP pg/L–mean (SD)	−3859.4 (−7700.3)
**Ultrasound Exam–At Admission**	***n* (%)**
Heart rhythm during ultrasound exam	
Sinusal rhythm	10 (33.3)
Atrial fibrillation	18 (60)
Atrial flutter	2 (6.7)
Inferior vena cava of >2.1 cm and < 50% of collapsability	12 (40.0)
Inferior vena cava of <2.1 cm and > 50% of collapsability	9 (30.0)
Lung score at admission–mean (SD)	16.5 (9.2)
Tricuspid regurgitation	
Moderate tricuspid regurgitation (>2.8 m/s and <3.4 m/s)–*n* (%)	6 (20.0)
Severe tricuspid regurgitation (>3.4 m/s)–*n* (%)	9 (30.0)
Pericardial effusion–*n* (%)	4 (13.3)
Low TAPSE (<16 mm)–*n* (%)	11 (36.7)
Mildly reduced ejection fraction (40–49%)–*n* (%)	5 (16.7)
Reduced ejection fraction (<40%)–*n* (%)	5 (16.7)
Probability of pulmonary hypertension *	
Low	7 (23.3)
Intermediate	12 (40.0)
High	11 (36.7)
Hepatic vein at admission	
S > D	1 (3.3)
S < D	17 (56.7)
S Reversal	11 (36.7)
Not measurable	1 (3.3)
Portal vein at admission	
Pulsatility < 30%	15 (23.3)
Pulsatility 30–50%	9 (40.0)
Pulsatility > 50%	6 (36.7)
Intra-renal vein at admission	
Continuous monophasic	13 (33.3)
Biphasic flow	13 (43.3)
Discontinuous monophasic	4 (13.3)
Femoral vein at admission	
Pulsatility < 30%	8 (26.7)
Pulsatility 30–50%	3 (10.0)
Pulsatility > 50%	19 (63.3)
**Ultrasound Exam–At Discharge**	***n* (%)**
Inferior vena cava of >2.1 cm and < 50% of collapsability	12 (40.0)
Inferior vena cava of <2.1 cm and > 50% of collapsability	9 (30.0)
Lung score at discharge–mean (SD)	9.3 (8.1)
Change in lung score–mean (SD)	6.7 (10.4)
Inferior vena cava of >2.1 cm and <50% of collapsability	8 (26.7)
Inferior vena cava of <2.1 cm and >50% of collapsability	10 (33.3)
Improve in inferior vena cava–*n* (%)	14 (46.7)
Hepatic vein at discharge	
S > D	9 (30.0)
S < D	11 (36.7)
S reversal	9 (30.0)
Not measurable	1 (3.3)
Improve in hepatic vein profile–*n* (%)	22 (73.3)
Portal vein at discharge	
Pulsatility < 30%	23 (76.3)
Pulsatility 30–50%	3 (10.0)
Pulsatility > 50%	4 (13.3)
Improve in portal vein profile–*n* (%)	9 (30.0)
Worsening in portal vein profile–*n* (%)	2 (6.7)
Intra-renal vein at discharge	
Continuous monophasic	17 (56.7)
Biphasic flow	9 (30.0)
Discontinuous monophasic	4 (13.3)
Improve in intra-renal vein profile–*n* (%)	6 (20.0)
Worsening in intra-renal vein profile–*n* (%)	4 (13.3)
Femoral vein at discharge	
Pulsatility < 30%	13 (43.3)
Pulsatility 30–50%	1 (3.3)
Pulsatility > 50%	16 (53.3)
Improve in femoral vein profile–*n* (%)	9 (30.0)
Worsening in femoral vein profile–*n* (%)	3 (10.0)
Improve in VE × US score–*n* (%)	9 (30.0)
Worsening in VE × US score–*n* (%)	7 (23.3)
VE × US score unchanged–*n* (%)	14 (46.7)
**Follow-Up**	
Length of stay–days (SD)EVEREST score at admission	9.1 (4.3) 10.1 (3.1)
EVEREST score at discharge	0.7 (0.8)
NYHA at admission	
NYHA I	1 (3.3)
NYHA II	9 (30.0)
NYHA III	18 (60.0)
NYHA IV	2 (6.7)
NYHA at discharge	
NYHA I	21 (70.0)
NYHA II	8 (26.7)
NYHA III	1 (3.3)
NYHA IV	0 (0.0)

NT-proBNP: NT-proB-type Natriuretic Peptide; NYHA: New York Heart Association; SD: standard deviation; VE × US: venous excess ultrasonography score. * According to the European guidelines for pulmonary hypertension [1].

**Table 2:** Improvement in ultrasound parameters at admission and discharge of patients included (*n* = 30).

Ultrasound Exam	At Admission	At Discharge	*p*-Value
Inferior vena cava of >2.1 cm and <50% of collapsibility–*n* (%)	12 (40.0)	12 (40.0)	0.132
Inferior vena cava of <2.1 cm and >50% of collapsibility–*n* (%)	9 (30.0)	9 (30.0)	0.132
Lung score–mean (SD)	16.5 (9.2)	9.3 (8.1)	<0.001
Hepatic vein (SD)	2.4 (0.6)	2.1 (0.9)	0.002
S > D–*n* (%)	1 (3.3)	9 (30.0)	
S < D–*n* (%)	17 (56.7)	11 (36.7)	
S reversal–*n* (%)	11 (36.7)	9 (30.0)	
Not measurable–*n* (%)	1 (3.3)	1 (3.3)	
Portal vein (SD)	1.7 (0.8)	1.4 (0.7)	0.023
Pulsatility < 30%–*n* (%)	15 (23.3)	23 (76.3)	
Pulsatility 30–50%–*n* (%)	9 (40.0)	3 (10.0)	
Pulsatility > 50%–*n* (%)	6 (36.7)	4 (13.3)	
Intra-renal vein (SD)	1.7 (0.7)	1.6 (0.7)	0.293
Continuous monophasic–*n* (%)	13 (33.3)	17 (56.7)	
Biphasic flow–*n* (%)	13 (43.3)	9 (30.0)	
Discontinuous monophasic–*n* (%)	4 (13.3)	4 (13.3)	
VE × US score (SD)	1.3 (1.0)	1.2 (1.1)	0.501
0–*n* (%)	8 (26.7)	10 (33.3)	
1–*n* (%)	11 (36.7)	9 (30.0)	
2–*n* (%)	6 (20.0)	7 (23.3)	
3–*n* (%)	5 (16.7)	4 (13.3)	
Femoral vein (SD)	2.4 (0.9)	2.1 (0.2)	0.161
Pulsatility < 30%–*n* (%)	8 (26.7)	13 (43.3)	
Pulsatility 30–50%–*n* (%)	3 (10.0)	1 (3.3)	
Pulsatility > 50%–*n* (%)	19 (63.3)	16 (53.3)	

NT-proBNP: NT-proB-type Natriuretic Peptide; SD: standard deviation; VE × US: venous excess ultrasonography score.

## Data Availability

The authors confirm that the data supporting the findings of this study are available from the corresponding author, upon reasonable request.
